# Self-reported attitudes and behaviours of medical students in Pakistan regarding academic misconduct: a cross-sectional study

**DOI:** 10.1186/1472-6939-15-43

**Published:** 2014-05-29

**Authors:** Kulsoom Ghias, Ghulam Rehmani Lakho, Hamna Asim, Iqbal Syed Azam, Sheikh Abdul Saeed

**Affiliations:** 1 Department of Biological and Biomedical Sciences, Aga Khan University, Stadium Road, P. O. Box 3500, Karachi 74800, Pakistan; 2 Department of Physiology, Rehman Medical College, Rehman Medical Institute, 4/A-3, Phase V, Hayatabad, Peshawar, Pakistan; 3 Medical College, Aga Khan University, Stadium Road, P. O. Box 3500, Karachi, Pakistan; 4 Department of Community Health Sciences, Aga Khan University, Stadium Road, P. O. Box 3500, Karachi, Pakistan; 5 College of Medicine, Mail code 6556, National Guards Health Affairs, King Saud bin Abdulaziz University, Jeddah 21423, Kingdom of Saudi Arabia

**Keywords:** Lying, Cheating, Stealing, Academic misconduct, Academic integrity, Medical students

## Abstract

**Background:**

Honesty and integrity are key attributes of an ethically competent physician. However, academic misconduct, which includes but is not limited to plagiarism, cheating, and falsifying documentation, is common in medical colleges across the world. The purpose of this study is to describe differences in the self-reported attitudes and behaviours of medical students regarding academic misconduct depending on gender, year of study and type of medical institution in Pakistan.

**Methods:**

A cross sectional study was conducted with medical students from one private and one public sector medical college. A pre-coded questionnaire about attitudes and behaviours regarding plagiarism, lying, cheating and falsifying documentation was completed anonymously by the students.

**Results:**

A total of 465 medical students filled the questionnaire. 53% of private medical college students reported that they recognize copying an assignment verbatim and listing sources as references as wrong compared to 35% of public medical college students. 26% of private medical college students self-report this behaviour as compared to 42% of public medical college students. 22% of private versus 15% of public medical college students and 21% of students in clinical years compared to 17% in basic science years admit to submitting a fake medical certificate to justify an absence. 87% of students at a private medical college believe that cheating in an examination is wrong as compared to 66% of public medical college students and 24% self-report this behaviour in the former group as compared to 41% in the latter. 63% of clinical year students identify cheating as wrong compared to 89% of their junior colleagues. 71% of male versus 84% of female respondents believe that cheating is wrong and 42% of males compared to 23% of females admit to cheating.

**Conclusions:**

There are significant differences in medical students’ attitudes and behaviours towards plagiarism, lying, cheating and stealing by gender, seniority status and type of institution. The ability to identify acts of academic misconduct does not deter students from engaging in the behaviour themselves, as evidenced by self-reporting.

## Background

Impeccable moral and ethical values are desired attributes of a medical practitioner. However, the medical profession today is facing a crisis of integrity as it moves from an ethics-based profession underpinned by core values of compassion and humanism to a more commercialized and business-like model driven by volumes and profits [1]. This change is felt even more acutely in developing countries, like Pakistan, where society at large and the medical profession in particular face major issues of governance and serious crises of integrity [2] as evidenced by frequent reports of unethical practices in clinical settings [3-5].

The desired competencies and moral practices of medical professionals are outcomes of academic training. Honesty and integrity are fundamental virtues expected from medical student. However academic misconduct, which includes but is not limited to cheating, plagiarism and falsifying documentation, is not uncommon in medical colleges across the world [6,7]. A number of studies point to the alarming rise in academic misconduct in educational institutions [8-10]. It has also been reported that students become desensitized to the academic misconduct as they advance in programs and accept cheating as a normal behaviour [10] and suggested that students indulging in cheating and dishonest behaviours may likely violate workplace ethics and indulge in dishonest practices with patients, peers and organizations later in their professional life [9].

Pakistan, a developing country, is currently facing challenges such as extreme poverty, vast financial, social and educational divides. Despite the brain drain and lack of competent faculty, there is a mushrooming of medical colleges and universities, both in the public and private sector. With limited resources, the focus has inexplicably shifted to quantity instead of quality and the critical issue of academic misconduct and student integrity in medical colleges has not been addressed adequately. A study on occurrence of academic misconduct has been reported in the literature from Pakistan, related to students of business, commerce and engineering [11] and another on knowledge and perceptions of plagiarism in the medical college fraternity [12]. The questionnaire-based study reported here was conducted to assess the prevailing self-reported attitudes and behaviours of medical students in Pakistan regarding academic misconduct, specifically lying, cheating and stealing, in addition to plagiarism, and to establish whether attitudes and behaviours vary depending on the gender, year of study and/or type of medical institution within Pakistan.

## Methods

### Study design

A cross sectional study was conducted in medical students from one private and one public sector medical college of Pakistan in 2010–2011.

### Questionnaire

The questionnaire used in this study was adapted from a previously reported study by Rennie and Crosby [7]. Minor terminology modifications were made to clarify and/or contextualize the questions and make them relevant to the study setting, but the questionnaire was not translated from the original English, as English is the language of instruction in the medical schools included in the study. The final questionnaire is a pre-coded version having 15 short scenarios related to plagiarism (6/15 scenarios), lying (including forging/falsifying official documents/data) (5/15 scenarios), cheating (2/15 scenarios) and stealing (2/15 scenarios). For each scenario presented (for example, “A student cheats in an examination”), respondents were asked whether a) the student in the scenario was wrong; and b) whether the respondent have ever done or would consider doing what is described in the scenario, to assess self-reported attitudes and behaviours, respectively. Respondents had the option to respond to each query with “Yes” or “No” or “Not sure”. The questionnaire also provided space after each scenario to add optional remarks or comments to justify the answers. Representative and/or revealing comments were selected by the authors for inclusion in this report.

### Data collection

An internal ethical review board is only active at the private medical college included in this study. Accordingly, ethical approval (#1611-BBS-ERC-2010 dated September 1, 2010) was received from the Ethical Review Committee of the former and formal permission taken from the Principal of the latter institution. Consent forms contained relevant details regarding the background and purpose of study, anonymity and confidentiality measures, possible benefits and risks, and right of refusal or withdrawal. All participants read and signed individual consent forms, which were returned with each completed questionnaire and filed by the study group. The study was administered through intermediary Year/rotation/department coordinators/faculty members who delivered the informed consent forms and questionnaires to the study participants in a regularly scheduled classes/curricular events and upon completion, collected the signed forms and anonymous questionnaires and delivered them to the study researchers. The target population of this study was students of one private and one public medical college. Students across all five years from the private institution were surveyed. Due to class and examination schedules, only students of Years 2 and 5 were available at the time of administration of this study in the public medical college. However, since these two years represent the two components of medical education i.e. basic science and clinical years, respectively, this sampling was deemed suitable.

### Statistical analysis

The percentage distribution of gender of students and mean with standard deviation of age of students by type of medical college was generated. The number and percentage of responses of students’ self-reported attitudes (indicating that the student in the scenario was wrong) and behaviours (indicating that the respondent has done or would consider doing what is described in the scenario) regarding academic misconduct were generated with 95% confidence intervals for their differences by type of medical college, class and gender. Crude and adjusted prevalence ratios for each scenario with 95% confidence intervals were computed using the algorithm of Cox-proportional hazard regression. Statistical Package for Social Sciences (SPSS; version 19.0) was used to analyze the data.

## Results

### Study sample

The study sampling is presented in Table 1. There was an overall response rate of 53% (260/489) from the private medical college, with the lowest response rate observed from students of Year 3 (25%) and 5 (28%). In comparison, there was an overall response rate of 41% (205/500) from students of Years 2 and 5 at a public medical college. 45% of all participants in the study were males, 41% in the private and 49% in the public medical college. Mean age of respondents at the private and public medical college was 20.2 years (SD = 1.8 years; range 17 to 26 years) and 21.5 years (SD = 1.9 years; range 18 to 26 years), respectively. More than half of all respondents (57%) were students in basic science years (i.e. Years 1 and 2 students in the private medical college and Year 2 students from the public medical college) and the remainder were students in clinical years (i.e. Years 3, 4 and 5 students in the private medical college and Year 5 students in the public medic college). More than two third (329/465; 70.5%) of all the students reported that they have studied ethics as a course in their institutions.

**Table 1:** Study sampling

	**Private medical college**				**Public medical college**			
**Year of study**	**Attending students**	**Questionnaires distributed**	**Questionnaires completed**	**Response rate (%)**	**Attending students**	**Questionnaires distributed**	**Questionnaires completed**	**Response rate (%)**
**1**^**a**^	97	97	74	76%	250	0	**-**	**-**
**2**^**a**^	99	99	92	93%	250	250	101	40%
**3**^**b**^	100	100	25	25%	250	0	**-**	**-**
**4**^**b**^	96	96	42	44%	250	0	**-**	**-**
**5**^**b**^	97	97	27	28%	250	250	104	42%

^a^Basic science year; ^b^Clinical science year.

### Plagiarism

In the first scenario related to plagiarism regarding a student who copies verbatim from the internet and other published sources and lists them as references, from the 452 students who stated their gender (202 males and 250 females), 204 (45%) responded that the student in the scenario is wrong, 86 (43%) male and 118 (47%) female (Table 2). 146 (33%) students out of the 438 that responded said that they had done or considered doing the same, 62 (32%) male and 84 (34%) female. From the 457 students who stated their class (264 in basic and 193 in clinical years), 207 (45%) answered that the student in this first scenario related to plagiarism is wrong, 120 (46%) students in basic science years and 87 (45%) students in clinical years. 86 (33%) students in basic science years and 60 (33%) students in clinical years responded that they had copied verbatim from the internet and other published sources and listed them as references or considered doing the same. From the 457 students from both medical colleges (259 from the private and 198 from the public medical college), 207 (45%) answered that the student in the scenario is wrong, 138 (53%) private and 69 (35%) public medical student (95% CI: 9, 28). 146 (33%) students out of the 443 students from both medical colleges that responded said that they had done or considered doing the same, 67 (26%) private and 79 (42%) public medical college students (95% CI: -25, -7). All remaining scenarios and results related to plagiarism are presented in Table 2 with significant differences at univariate level in bold.

**Table 2:** Univariate analysis of student self-reported attitudes and behaviours related to plagiarism by gender, class (basic, clinical) and type of medical college (private, public)*

**Scenarios related to plagiarism**	**Male**	**Female**	**95% CI**	**Basic**	**Clinical**	**95% CI**	**Private**	**Public**	**95% CI**
**For an assignment, a student copies verbatim (word-for-word) from the internet and other published sources (textbooks, papers) and lists them as references.**									
a. The student is wrong.	86/202 (43)	118/250 (47)	-14, 5	120/264 (46)	87/193 (45)	-9, 10	138/259 (53)	69/198 (35)	**9, 28**
b. Have done or would consider doing the same.	62/438 (32)	84/245 (34)	-11, 7	86/260 (33)	60/183 (33)	-9, 9	67/256 (26)	79/187 (42)	**-25, -7**
**For an assignment, a student copies from the internet and other published sources (textbooks, papers) without acknowledging the sources.**									
a. The student is wrong.	149/202 (74)	209/251 (83)	**-17, -2**	215/265 (81)	146/193 (76)	-2, 13	226/259 (87)	135/199 (68)	**12, 27**
b. Have done or would consider doing the same.	47/200 (24)	35/248 (14)	**2, 17**	40/262 (15)	42/191 (22)	-14, 1	35/258 (14)	47/195 (24)	**-18, -3**
**For an assignment, a student copies from assignments submitted earlier by senior peers.**									
a. The student is wrong.	149/199 (75)	199/251 (79)	-12, 4	211/265 (80)	141/190 (74)	-3, 13	193/258 (75)	159/197 (81)	-14, 2
b. Have done or would consider doing the same.	50/184 (27)	41/237 (17)	**2, 18**	33/261 (13)	59/165 (36)	**-32, -15**	58/256 (23)	34/170 (20)	-5, 11
**A student helps a friend by writing an assignment for him/her.**									
a. The student is wrong.	102/202 (51)	119/251 (47)	-6, 12	145/266 (55)	80/192 (42)	**4, 22**	134/257 (52)	91/201 (45)	-2, 16
b. Have done or would consider doing the same.	97/202 (48)	129/245 (53)	-14, 5	123/261 (47)	104/191 (55)	-17, 2	111/254 (44)	116/198 (59)	**-24, -6**
**A student lends his work to a friend to copy.**									
a. The student is wrong.	96/203 (47)	132/251 (53)	-15, 4	160/265 (60)	70/195 (36)	**16, 34**	115/256 (45)	115/204 (56)	**-21, -2**
b. Have done or would consider doing the same.	70/203 (35)	112/247 (45)	**-20, -2**	102/262 (39)	83/193 (43)	-13, 5	129/253 (51)	56/202 (28)	**15, 32**
**A student copies a friend’s work without telling him.**									
a. The student is wrong.	175/202 (87)	235/251 (94)	**-13, -1**	240/264 (91)	175/195 (90)	-4, 7	232/255 (91)	183/204 (90)	-4, 7
b. Have done or would consider doing the same.	20/200 (10)	11/247 (5)	**1, 11**	12/259 (5)	19/193 (10)	**-10, -0.3**	15/253 (6)	16/199 (8)	-7, 3
**A student re-submits the same report for another part of the course.**									
a. The student is wrong.	136/201 (68)	163/249 (66)	-7, 11	178/262 (68)	125/194 (64)	-5, 12	150/254 (59)	153/202 (76)	**-25, -8**
b. Have done or would consider doing the same.	33/200 (17)	29/244 (12)	-2, 11	28/257 (11)	34/191 (18)	**-14, -0.2**	41/251 (16)	21/197 (11)	-0.6, 12

*Agreement with the scenario stems i.e. a response of “Yes”, is reported here in absolute numbers out of total number of respondents in the sub-group and corresponding percentages. Remaining responses were either “No” or “Not sure”. Not all respondents provided an answer to each scenario/question. Significant differences are in bold text.

In the multivariable analysis, four explanatory variables i.e. gender, class, type of medical college and whether ethics is taught at the institution were considered for each scenario related to plagiarism (Table 3). In the scenario regarding a student who copies verbatim from the internet and other published sources and lists them as references, the answer that the questionnaire respondents had done or considered doing the same was lower among students of the private medical college as compared to public medical college students (Adjusted prevalence ratio (APR) = 0.6; 95% CI: 0.4, 0.9), when adjusted for the other three explanatory variables. Similar finding was observed in private medical college students as compared to public medical college students (APR = 0.5; 95% CI: 0.3, 0.9) for the scenario about a student who copies from the internet and other published sources without acknowledging the sources. In the scenario regarding a student lending his work to a friend to copy, the response that the student in the scenario is wrong was significantly lower for students in clinical years as compared to basic sciences years (APR = 0.6; 95% CI: 0.4, 0.8), when adjusted for the other three explanatory variables. Similar finding for the same scenario was observed for private medical students as compared to public medical students (APR = 0.7; 95% CI: 0.5, 0.9). Whether the questionnaire respondent had done or considered doing the same is significantly higher among students in the clinical years as compared to basic sciences years (APR = 1.4; 95% CI: 1.1, 2) and significantly lower among those students who reported that they studied ethics as a course as compared to those who had not (APR = 0.5; 95% CI: 0.3, 0.8). Other significant findings are provided in bold in Table 3.

**Table 3:** Multivariate analysis of student self-reported attitudes and behaviours related to plagiarism*

**Explanatory variables**	**Female**		**Clinical**		**Private medical college**		**Formally studied ethics**	
**Scenarios related to plagiarism**	**CPR (95% CI) APR (95% CI)**		**CPR (95% CI) APR (95% CI)**		**CPR (95% CI) APR (95% CI)**		**CPR (95% CI) APR (95% CI)**	
**For an assignment, a student copies verbatim (word-for-word) from the internet and other published sources (textbooks, papers) and lists them as references.**								
a. The student is wrong.	1.1 (0.8, 1.5)	1.1 (0.8, 1.4)	1 (0.8, 1.3)	1.1 (0.8, 1.5)	**1.5 (1.2, 2.0)**	1.4 (0.97, 1.9)	**0.7 (0.5, 0.9)**	0.8 (0.5, 1.2)
b. Have done or would consider doing the same.	1.1 (0.8, 1.5)	1.1 (0.8, 1.5)	1 (0.7, 1.4)	1 (0.7, 1.4)	**0.6 (0.5, 0.9)**	**0.6 (0.4, 0.9)**	1.2 (0.8, 1.7)	0.9 (0.6, 1.4)
**For an assignment, a student copies from the internet and other published sources (textbooks, papers) without acknowledging the sources.**								
a. The student is wrong.	1.1 (0.9,1.4)	1.1 (0.9, 1.4)	0.9 (0.8, 1.2)	1 (0.8, 1.2)	**1.3 (1.0, 1.6)**	1.3 (1.0, 1.7)	0.9 (0.7, 1.1)	1.0 (0.8, 1.4)
b. Have done or would consider doing the same.	**0.6 (0.4, 0.96)**	0.6 (0.4, 1.01)	1.2 (0.9, 2.2)	1.4 (0.8, 2.2)	**0.6 (0.4, 0.9)**	**0.5 (0.3, 0.9)**	1.3 (0.8, 2.0)	0.8 (0.4,1.4)
**For an assignment, a student copies from assignments submitted earlier by senior peers.**								
a. The student is wrong.	1.1 (0.9, 1.3)	1.1 (0.9, 1.3)	0.9 (0.8, 1.2)	0.9 (0.7, 1.1)	0.9 (0.8, 1.1)	0.97 (0.8, 1.2)	1.1 (0.9, 1.4)	1.1 (0.9, 1.5)
b. Have done or would consider doing the same.	**0.6 (0.4, 0.96)**	0.7 (0.5, 1.1)	**2.8 (1.9, 4.3)**	**2.9 (1.8, 4.5)**	1.1 (0.7, 1.7)	1.1 (0.7, 1.8)	1 (0.6, 1.6)	0.8 (0.4, 1.4)
**A student helps a friend by writing an assignment for him/her.**								
a. The student is wrong.	0.9 (0.7, 1.2)	0.9 (0.7, 1.2)	0.8 (0.6, 1.0)	0.8 (0.6, 1.0)	1.2 (0.9, 1.5)	1.1 (0.8. 1.5)	0.8 (0.6, 1.1)	1 (0.7, 1.4)
b. Have done or would consider doing the same.	1.1 (0.8, 1.4)	1.2 (0.9, 1.5)	1.2, (0.9, 1.5)	1.1 (0.9, 1.5)	**0.8 (0.6, 0.97)**	0.8 (0.6, 1.1)	1.3 (1, 1.7)	1 (0.7, 1.5)
**A student lends his work to a friend to copy.**								
a. The student is wrong.	1.1 (0.9, 1.5)	1.0 (0.8, 1.3)	**0.6 (0.5, 0.8)**	**0.6 (0.4, 0.8)**	0.8 (0.6, 1.0)	**0.7 (0.5, 0.9)**	0.9 (0.6, 1.2)	0.9 (0.6, 1.3)
b. Have done or would consider doing the same.	1.3 (0.98, 1.8)	1.3 (0.95 1.7)	1.1 (0.8, 1.5)	**1.4 (1.1, 1.95)**	1.8 (1.3, 2.5)	1.3 (0.9, 1.9)	**0.5 (0.3, 0.7)**	**0.5 (0.3, 0.8)**
**A student copies a friend’s work without telling him.**								
a. The student is wrong.	1.1 (0. 9, 1.3)	1.1 (0.9, 1.3)	1 (0.8, 1.2)	1 (0.8, 1.2)	1.0 (0.8, 1.2)	1.0 (0.8, 1.3)	1.0 (0.8, 1.3)	1.0 (0.8, 1.4)
b. Have done or would consider doing the same.	**0.5 (0.2, 0.9)**	0.5 (0.2, 1.1)	**2.1 (1.0, 4.4)**	2.2 (1, 4.8)	0.7 (0.4, 1.5)	0.7 (0.3, 1.5)	1.1 (0.5, 2.3)	0.5 (0.2, 1.4)
**A student re-submits the same report for another part of the course.**								
a. The student is wrong.	1 (0.8, 1.2)	1 (0.8, 1.2)	1 (0.8, 1.2)	0.8 (0.6, 1.1)	**0.8 (0.6, 0.98)**	0.9 (0.7, 1.2)	**1.3 (1.0, 1.7)**	1.3 (0.95, 1.8)
b. Have done or would consider doing the same.	0.7 (0.4, 1.2)	0.8 (0.5, 1.2)	1.6 (0.99, 2.7)	1.7 (0.99, 2.9)	1.5 (0.9, 2.6)	1.8 (0.9, 3.6)	0.9 (0.5, 1.6)	1.0 (0.5, 2.2)

*Agreement with the scenario stems i.e. a response of “Yes”, is reported here in absolute numbers out of total number of respondents in the sub-group and corresponding percentages. Remaining responses were either “No” or “Not sure”. Not all respondents provided an answer to each scenario/question. Significance is indicated by bold text.

### Lying

In the first scenario related to lying regarding a student who omits and/or adds data points to show the desired results when plotting a graph for an experiment, from the 450 students who stated their gender (202 males and 248 females), 289 (64%) responded that the student in the scenario is wrong, 129 (64%) male and 160 (65%) female (Table 4). 168 (37%) students out of the 450 that responded said that they had done or considered doing the same, 81 (40%) male and 87 (35%) female. From the 456 students who stated their class (260 in basic and 196 in clinical years), 293 (64%) answered that the student in this first scenario related to lying is wrong, 171 (66%) students in basic science years and 122 (62%) students in clinical years. 92 (36%) students in basic science years and 77 (39%) students in clinical years responded that they had edited data points in graphs to show the desired results or considered doing the same. From the 456 students from both medical colleges (258 from the private and 198 from the public medical college), 293 (64%) answered that the student in the scenario is wrong, 200 (78%) private and 93 (47%) public medical students (95% CI: 23, 39). 169 (37%) students out of the 456 students from both medical colleges that responded said that they had done or considered doing the same, 75 (29%) private and 94 (48%) public medical college students (95% CI: -27, -10). All remaining scenarios and results related to lying are presented in Table 4 with significant differences at univariate level in bold.

**Table 4:** Univariate analysis of student self-reported attitudes and behaviours related to lying by gender, class (basic, clinical) and type of medical college (private, public)*

**Scenarios related to lying**	**Male**	**Female**	**95% CI**	**Basic**	**Clinical**	**95% CI**	**Private**	**Public**	**95% CI**
**While plotting a graph for an experiment, a student omits and/or adds data points to show the desired results.**									
a. The student is wrong.	129/202 (64)	160/248 (65)	-10, 8	171/260 (66)	122/196 (62)	-5, 12	200/258 (78)	93/198 (47)	**22, 39**
b. Have done or would consider doing the same.	81/204 (40)	87/246 (35)	-5, 1	92/259 (36)	77/197 (39)	-13, 5.4	75/258 (29)	94/198 (48)	**-27, -10**
**A student writes “Examination – normal” in his patient presentation when he has not performed the procedure.**									
a. The student is wrong.	176/200 (88)	221/252 (88)	-6, 6	230/263 (88)	171/195 (88)	-6, 6	232/254 (91)	169/204 (83)	**2, 15**
b. Have done or would consider doing the same.	56/201 (28)	31/245 (13)	**8, 23**	20/257 (8)	68/193 (35)	**-35, -20**	25/251 (10)	63/199 (32)	**-29, -14**
**A student fakes an illness to justify an absence.**									
a. The student is wrong.	131/201 (65)	182//251 (73)	-16, 1	199/263 (76)	118/195 (61)	**7, 24**	173/255 (68)	144/203 (71)	-12, 6
b. Have done or would consider doing the same.	38/201 (19)	55/249 (22)	-11, 4.4	55/261 (21)	38/194 (20)	-6, 9	79/253 (31)	14/202 (7)	**17, 31**
**A student submits a fake medical certificate to justify an absence.**									
a. The student is wrong.	128/201 (64)	175/251 (70)	-15, 3	192/264 (73)	115/194 (59)	**5, 22**	176/255 (69)	131/203 (65)	-4, 13
b. Have done or would consider doing the same.	40/199 (20)	45/246 (18)	-6, 9	45/260 (17)	40/189 (21)	-11, 4	56/250 (22)	29/199 (15)	**1, 15**
**A student forges a professor’s signature on a piece of work.**									
a. The student is wrong.	155/199 (78)	214/251 (85)	**-15, -0.1**	233/263 (89)	140/193 (73)	**9, 24**	241/255 (95)	132/201 (66)	**22, 36**
b. Have done or would consider doing the same.	17/198 (9)	18/249 (7)	-4, 6	20/260 (8)	15/192 (8)	-5, 5	16/253 (6)	19/199 (10)	-8, 2

*Agreement with the scenario stems i.e. a response of “Yes”, is reported here in absolute numbers out of total number of respondents in the sub-group and corresponding percentages. Remaining responses were either “No” or “Not sure”. Not all respondents provided an answer to each scenario/question. Significant differences are in bold text.

The multivariable analysis for each scenario related to lying is shown in Table 5. In the scenario regarding a student who while plotting a graph for an experiment omits and/or adds data points to show the desired results, the response that the student in the scenario is wrong was significantly higher among private medical college students as compared to public medical college students (APR = 1.5; 95% CI: 1.1, 1.9) when adjusted for the other three explanatory variables. Whether the questionnaire respondent had done or considered doing the same is significantly lower among private medical college students as compared to public medical college students (APR = 0.7; 95% CI: 0.5, 1). In the scenario regarding a student who writes “Examination – normal” in his patient presentation when he has not performed the procedure, the answer that the respondent had done or considered doing the same is significantly higher in clinical year students as compared to basic sciences students (APR = 3.3; 95% CI: 1.9, 5.7) and lower in private medical college students as compared to public medical college students (APR = 0.4; 95% CI: 0.3,0.8). In the scenario where a student fakes an illness to justify an absence, whether the respondent had done or considered doing the same is significantly higher among private medical college students as compared to public medical college student (APR = 4.2; 95% CI: 2.3, 9) when adjusted for other three explanatory variables. Other significant findings are provided in bold in Table 5.

**Table 5:** Multivariate analysis of student self-reported attitudes and behaviours related to lying*

**Explanatory variables**	**Female**		**Clinical**		**Private medical college**		**Formally studied ethics**	
**Scenarios related to plagiarism**	**CPR (95% CI) APR (95% CI)**		**CPR (95% CI) APR (95% CI)**		**CPR (95% CI) APR (95% CI)**		**CPR (95% CI) APR (95% CI)**	
**While plotting a graph for an experiment, a student omits and/or adds data points to show the desired results.**								
a. The student is wrong.	1.0 (0.8, 1.3)	1 (0.8, 1.2)	1 (0.8, 1.2)	1.1 (0.9, 1.4)	**1.7 (1.3, 2.1)**	**1.5 (1.1, 1.9)**	**0.6 (0.5, 0.8)**	0.7 (0.5, 1.0)
b. Have done or would consider doing the same.	0.9 (0.7, 1.2)	0.9 (0.7, 1.3)	1.1 (0.8, 1.5)	0.95 (0.7, 1.3)	**0.6 (0.5, 0.8)**	**0.7 (0.5, 0.98)**	**1.5 (1.1, 2.0)**	1.2 (0.8, 1.8)
**A student writes “Examination – normal” in his patient presentation when he has not performed the procedure.**								
a. The student is wrong.	1 (0.8, 1.2)	1 (0.8, 1.2)	1.0 (0.8, 1.2)	1 (0.8, 1.2)	1.1 (0.9, 1.3)	1.2 (0.9, 1.5)	1.0 (0.8, 1.3)	1.2 (0.9, 1.6)
b. Have done or would consider doing the same.	**0.5 (0.3, 0.7)**	0.7 (0.4, 1.0)	**4.5 (2.8, 7.5)**	**3.3 (1.9, 5.7)**	**0.3 (0.2, 0.5)**	**0.4 (0.3, 0.8)**	**3.1 (2, 4.7)**	1.2 (0.7, 2.1)
**A student fakes an illness to justify an absence.**								
a. The student is wrong.	1.1 (0.9, 1.4)	1.1 (0.9, 1.3)	0.8 (0.6, 1.0)	0.9 (0.7, 1.1)	0.96 (0.8, 1.2)	0.8 (0.6, 1.1)	0.8 (0.6, 1.1)	0.8 (0.6, 1.1)
b. Have done or would consider doing the same.	1.2 (0.8, 1.8)	1.1 (0.7, 1.6)	0.9 (0.6, 1.4)	1.2 (0.8, 1.8)	**4.5 (2.6, 8)**	**4.5 (2.3, 9)**	**0.4 (0.2, 0.7)**	0.9 (0.5, 2)
**A student submits a fake medical certificate to justify an absence.**								
a. The student is wrong.	1.1 (0.9, 1.4)	1.1 (0.8, 1.3)	0.8 (0.7, 1.0)	0.9 (0.7, 1.1)	1.1 (0.9, 1.3)	0.9 (0.7, 1.2)	**0.8 (0.6, 0.99)**	0.8 (0.6, 1.1)
b. Have done or would consider doing the same.	0.9 (0.6, 1.4)	0.9 (0.6, 1.4)	1.2 (0.8, 1.9)	1.4 (0.9, 2.2)	1.5 (0.98, 2.4)	1.4 (0.8, 2.5)	0.7 (0.4, 1.2)	0.7 (0.4, 1.5)
**A student forges a professor’s signature on a piece of work.**								
a. The student is wrong.	1.1 (0.9, 1.4)	1.0 (0.8, 1.3)	0.8 (0.7, 1.0)	0.9 (0.7, 1.2)	**1.4 (1.2, 1.8)**	1.3 (0.98, 1.6)	**0.6 (0.5, 0.8)**	0.8 (0.6, 1.1)
b. Have done or would consider doing the same.	0.8 (0.4, 1.6)	0.8 (0.4, 1.7)	1.0 (0.5, 2)	0.9 (0.4, 1.8)	0.7 (0.3, 1.3)	0.8 (0.3, 1.8)	1.5 (0.8, 2.9)	1.3 (0.5, 3.3)

*Agreement with the scenario stems i.e. a response of “Yes”, is reported here in absolute numbers out of total number of respondents in the sub-group and corresponding percentages. Remaining responses were either “No” or “Not sure”. Not all respondents provided an answer to each scenario/question. Significance is indicated by bold text.

### Cheating and stealing

In the first scenario related to cheating and stealing regarding a student who cheats in an examination, from the 456 students who stated their gender (205 males and 251 females), 356 (78%) responded that the student in the scenario is wrong, 145 (71%) male and 211 (84%) female (95% CI: -21, -6) (Table 6). 142 (31%) students out of the 453 that responded said that they had done or considered doing the same, 85 (42%) male and 57 (23%) female (95% CI: 11, 28). From the 462 students who stated their class (266 in basic and 196 in clinical years), 360 (78%) answered that the student in this first scenario related to cheating is wrong, 237 (89%) students in basic science years and 123 (63%) students in clinical years (95% CI: 19, 34). 63 (24%) students in basic science years and 81 (41%) students in clinical years responded that they had cheated in an examination or considered cheating (95% CI: -26, -8). From the 462 students from both medical colleges (260 from the private and 202 from the public medical college), 360 (78%) answered that the student in the scenario is wrong, 227 (87%) private and 133 (66%) public medical students (95% CI: 14, 29). 144 (31%) students out of the 459 students from both medical colleges that responded said that they had cheated in an examination or considered doing the same, 62 (24%) private and 82 (41%) public medical college students (95% CI: -26, -8). All remaining scenarios and results related to cheating and stealing are presented in Table 6 with significant differences at univariate level in bold.

**Table 6:** Univariate analysis of student self-reported attitudes and behaviours related to cheating and stealing by gender, class (basic, clinical) and type of medical college (private, public)*

**Scenarios related to cheating and stealing**	**Male**	**Female**	**95% CI**	**Basic**	**Clinical**	**95% CI**	**Private**	**Public**	**95% CI**
**A student cheats in an examination.**									
a. The student is wrong.	145/205 (71)	211/251 (84)	**-21, -6**	237/266 (89)	123/196 (63)	**19, 34**	227/260 (87)	133/202 (66)	**14, 29**
b. Have done or would consider doing the same.	85/203 (42)	57/250 (23)	**11, 28**	63/262 (24)	81/197 (41)	**-26, -8**	62/259 (24)	82/200 (41)	**-26, -8**
**A student reports that another student was cheating during an examination.**									
a. The student is wrong.	103/206 (50)	101/252 (40)	**1, 19**	93/266 (35)	115/198 (58)	**-32, -14**	107/259 (41)	101/205 (49)	-17, 1
b. Have done or would consider doing the same.	30/205 (15)	34/248 (14)	-6, 7	42/263 (16)	24/196 (12)	-3, 10	37/258 (14)	29/201 (14)	-7, 6
**A model goes missing from the Anatomy lab and a student who is aware of the culprit reports the information to the concerned faculty/staff.**									
a. The student is wrong.	47/206 (23)	46/252 (18)	-3, 12	50/266 (19)	45/198 (23)	-12, 3.6	54/260 (21)	41/204 (20)	-7, 8
b. Have done or would consider doing the same.	55/206 (27)	71/249 (29)	-10, 7	85/263 (32)	42/198 (21)	**3, 19**	91/259 (35)	36/202 (18)	**9, 25**

^*^Agreement with the scenario stems i.e. a response of “Yes”, is reported here in absolute numbers out of total number of respondents in the sub-group and corresponding percentages. Remaining responses were either “No” or “Not sure”. Not all respondents provided an answer to each scenario/question. Significant differences are in bold text.

The multivariable analysis for each scenario related to cheating and stealing is shown in Table 7. In the scenario regarding a student who cheats in an examination, the response that the student is wrong is significantly lower among clinical year students as compared to basic sciences students (APR = 0.8; 95% CI: 0.6, 0.99) when adjusted for the other three explanatory variables. Whether respondents had done or considered doing the same is significantly lower in females as compared to male students (APR = 0.6; 95% CI: 0.4, 0.9). In another scenario in which a student reports that another student was cheating during the examination, the response that the student is wrong is significantly higher among clinical year students as compared to basic sciences students (APR = 1.6; 95% CI: 1.2, 2.2) when adjusted for the other three explanatory variables. Other significant findings are provided in bold in Table 7.

**Table 7:** Multivariate analysis of student self-reported attitudes and behaviours related to cheating and stealing*

**Explanatory variables**	**Female**		**Clinical**		**Private medical college**		**Formally studied ethics**	
**Scenarios related to plagiarism**	**CPR (95% CI) APR (95% CI)**		**CPR (95% CI) APR (95% CI)**		**CPR (95% CI) APR (95% CI)**		**CPR (95% CI) APR (95% CI)**	
**A student cheats in an examination.**								
a. The student is wrong.	1.2 (0.96, 1.5)	1.1 (0.9, 1.4)	**0.7 (0.6, 0.9)**	**0.8 (0.6, 0.99)**	**1.3 (1.1, 1.6)**	1.1, (0.9, 1.5)	**0.6 (0.5, 0.8)**	0.8 (0.6, 1.1)
b. Have done or would consider doing the same.	**0.6 (0.4, 0.8)**	**0.6 (0.4, 0.9)**	**1.7 (1.2, 2.4)**	1.3 (0.9, 1.9)	**0.6 (0.4, 0.8)**	0.8 (0.5, 1.2)	**1.98 (1.4, 2.7)**	1.4 (0.9, 2.2)
**A student reports that another student was cheating during an examination.**								
a. The student is wrong.	0.8 (0.6, 1.1)	0.9 (0.7, 1.2)	**1.7 (1.3, 2.2)**	**1.6 (1.2, 2.2)**	0.8 (0.6, 1.1)	0.9 (0.7, 1.3)	**1.3 (1.0, 1.8)**	1.0 (0.7, 1.5)
b. Have done or would consider doing the same.	0.9 (0.6, 1.5)	0.9 (0.5, 1.4)	0.8 (0.5, 1.3)	0.8 (0.4, 1.3)	1 (0.6, 1.6)	0.8 (0.5, 1.4)	0.7 (0.4, 1.3)	0.7 (0.4, 1.4)
**A model goes missing from the Anatomy lab and a student who is aware of the culprit reports the information to the concerned faculty/staff.**								
a. The student is wrong.	0.8 (0.5, 1.2)	0.8 (0.5, 1.3)	1.2 (0.8, 1.8)	1.2 (0.8, 1.8)	1.0 (0.7, 1.6)	1.1 (0.7, 1.8)	1.1 (0.7, 1.7)	1.1 (0.6, 1.9)
b. Have done or would consider doing the same.	1.1 (0.8, 1.5)	1 (0.7, 1.4)	**0.7 (0.5, 0.95)**	0.8 (0.5, 1.1)	**1.97 (1.3, 2.9)**	**1.6 (1, 2.5)**	**0.5 (0.3, 0.7)**	0.6 (0.4, 1.2)

*Agreement with the scenario stems i.e. a response of “Yes”, is reported here in absolute numbers out of total number of respondents in the sub-group and corresponding percentages. Remaining responses were either “No” or “Not sure”. Not all respondents provided an answer to each scenario/question. Significant differences are in bold text.

## Discussion

Both private and public medical colleges in Pakistan enroll students, at the average age of 18 years, into five year undergraduate programmes after completion of higher secondary education. At the private medical college in this study, approximately one hundred students from diverse educational backgrounds are enrolled each year at an admission ratio of 1:40. Entering students hail from the British GCE system and the local Board of Intermediate Education, among others. The medical curriculum is clinically contextual, integrated and spiral, with a formal ethics/bioethics curriculum included as part of the longitudinal themes that run throughout the five years [13]. In comparison, public medical colleges tend to have larger classes of on average 250 students in each year, as in the case of the institution surveyed in this study. Students hail almost exclusively from the same educational background, that is, the local Board of Intermediate Studies. The five year programme at the public medical college in this study has no formal ethics curriculum. Both medical colleges in this study are among the 90 institutions recognized by the Pakistan Medical and Dental Council, a national regulatory authority, which recognizes 52 colleges in the private sector and 38 colleges in the public sector [14].

In our study, we found differences in self-reported attitudes and behaviours of students in private versus public medical colleges, junior versus senior students and male versus female students. Lack of integrity among medical school applicants, students, residents and even physicians has been widely reported [15-18], with differences in attitudes and behaviours and a decline in ethical behaviour across years [10,19].

Students who are able to identify acts of plagiarism appreciate the importance of not copying and citing sources as is evident by a student comment:

“[Plagiarism] hinders the creativity and learning ability of the student”

Comments from respondents who do not believe it is wrong to copy from sources reveal a lack of understanding of the act and the seriousness of plagiarism, an ignorance which may stem from the lack of a formal ethics curriculum:

“[If] listing it as the reference, there is nothing wrong [in copying verbatim]. Resources are there to be copied.”

“If an extensive research is done, sometimes it is not possible to cite all your sources.”

Indeed, a previous study assessing the knowledge and perceptions of medical students and faculty members in Pakistan reported a general lack of information [12] despite the fact that plagiarism is an issue of growing concern in the academic community [20] and lay press [21].

Justification for committing plagiarism is based on perception of importance and consequences of the assignment:

“When there is a dire need to submit a very important assignment on which [one’s] future depends, morals might be compromised”

“Certain assignments are just a burden to complete on time [without plagiarism] and promise no clinical learning”

Plagiarizing from peers is justified on the grounds of friendship and saving time:

“Friends are the true medicine of life.”

“If a student has done a great job [earlier], why not copy it? As long as the copying student understands it anyway.”

“Some assignments are just work. Busy is not conducive to medical college.”

Students in Years 1 and 2 are more likely to identify acts of plagiarism and less likely to have committed or considered committing them. The reasons for this are probably multifactorial. Awareness of acts of academic misconduct, such as plagiarism, is raised in earlier years as part of a formal ethics curriculum, with a shift in curricular focus to clinical ethics issue and patient rights in later years. This may counter-act the fact that habits are likely inculcated in earlier schooling and through societal norms [22] and that students come to medical college “prepared to cheat” [23]. Stress may be another factor leading to differences in behaviour. Previous studies have reported high prevalence rate of stress among medical students [24] due to academic stressors such as performance, evaluation and curricular volume [25,26], which increase with each passing year. In general, stress and lack of time may contribute to students’ willingness to indulge in plagiarism. Unfortunately, it is also possible that one reason for a difference in behaviour between junior and senior students is that the juniors have not yet had the “opportunity” to commit plagiarism, which may present as the work load increases. Interestingly, although several students do not identify copying from published sources as plagiarism (“*Resources are meant to be shared.”*), a large percentage believes it is wrong to plagiarize a friend’s work without their knowledge (*“I would never want my work to be copied without reference, so, wouldn’t do it to others”*).

Believing that lying is acceptable and admitting to the same is an alarming trend exhibited in this study. Although a large majority of both private and public medical college students identified that a student is wrong to write “Examination – normal” in his patient presentation when he has not performed the procedure, almost a third of public medical college students admit to doing or considering doing this. Dishonesty in patient care activities, such as recording tasks not performed, reporting findings elicited by others, and lying about having ordered a test, are often motivated by fear and a desire to appear knowledgeable [17]. This may be a contributing factor in this study as well, along with poor time management which may compel students to make false claims. In the case of faking illness or submitting fake medical certificates to justify absences, not only do students admit to the behaviour, they place the blame squarely on the shoulders of the administration:

“Because the administration doesn’t give leave except for illness, the students are forced to lie.”

There are indeed strict attendance policies at the surveyed private institution predicated on the fact that attendance is essential for learning, particularly in a skills-based profession such as medicine, which explains why greater number of students at the private medical college and particularly in senior years admit to this behaviour. Although the students’ justification does not absolve them of lying, it would be worthwhile for institutions to revisit medical leave policies for more innovative solutions to resolve this issue [27].

It has previously been reported that students who have cheated earlier during their primary or secondary schooling were more likely to carry on with similar behaviours than those who had never done so [16]. In Pakistan, although cheating is not universally uncommon, it is more prevalent in the local school system from students in the public sector invariably hail. Certainly the oft-cited justification *“everyone does it”* indicates that cheating is widespread and as an act, has repeat-offenders. Indeed, it has been previously reported that cheating may be influenced by peer acceptance of this behavior [28] and that students cite illness, workload, and perception of the material taught as “trivial” as reasons for cheating [17]. Not surprisingly, similar justifications were provided in this study. Additionally, students again blamed the system and made a distinction on the basis of gravity of the exam:

“[Cheating] not always wrong. Sometimes the exams are pointless and the system is flawed.”

“Depends [on] if it was a fair paper in the first place.”

“If [the student] copies word [for] word, then he is wrong, and in major exams. But, in tests its ok.”

When it comes to reporting acts of cheating or stealing, it is apparent that the respondents grapple with a moral dilemma. Although the students in the study accept that reporting such acts is correct (*“By reporting the cheating, the student is actually helping the other student for his/her better[ment]”*), very few admit to having done the same due to demands on loyalty and fear of consequences:

“I know it is the right thing to do, but social problems then arise.”

“Student unity [supersedes reporting]”

“[Better to] mind your own business”

Indeed, previous studies have reported that students are not likely to report because of fear of repercussions by their peers, being outcast and believing that it is not a student’s responsibility to report the misconduct of others [29,30]. Interestingly, a previous study revealed that female are more lenient regarding penalties for unprofessional behaviour [31], which may influence attitudes and behaviours related to reporting peers who have cheated or stolen. However, our study did not reveal any gender differences with regards to reporting.

On the other hand, our study found differences in other attitudes and behaviours between males and females, with the latter group better at identifying acts of plagiarism and less likely to lie or cheat. Previous studies have reported that females have a better understanding of academic integrity and are less likely to indulge in acts of academic misconduct as compared to males [6,16,32]. Generally, females tend to be higher academic achievers in Pakistani medical colleges, which may explain why they are less likely to indulge in acts of academic misconduct. Also, as previously discussed, there is a correlation with past behaviour [16] and it has been reported that females are less likely to exhibit delinquent behaviour in secondary school [32,33].

The main limitation of the study is that it was conducted in just one private and one public sector medical college in Pakistan, which limited its scope as a representative sample. However, as such, it has served as a pilot study in this research area. Further representative studies will be conducted in the future with appropriate sample size and additional independent factors like the role of socio-economic status [34], religion and religiosity [35] on academic integrity.

## Conclusions

In our study significant differences were observed in the self-reported attitudes and behaviours towards academic misconduct between medical students of private and public sector institutions, between junior and senior students and in certain scenarios, between males and females. The students surveyed can identify what constitutes lying, cheating and stealing, but not all forms of plagiarism. However, ability to identify acts of academic misconduct does not deter students from engaging in the behaviour themselves. Academic integrity is essential in preparing medical students to be ethical and purposeful citizens of the profession and the world. It is therefore critical that institutions make concerted to generate a culture of integrity to correct student behaviours that may have previously been condoned in early educational environments and within the society at large [22]. These efforts may include, but are not limited to, development and implementation of formal ethics curricula, attention to the hidden curriculum [36], exposure to good role modeling [37], establishment and communication of sound academic integrity policies [38].

## Competing interests

The authors declare they have no competing interests.

## Authors’ contributions

KG conceived of the study, facilitated data collection, participated in data analysis and interpretation, and preparation of the manuscript. GRL conceived of the study, facilitated data collection, participated in data analysis and interpretation, and preparation of the manuscript. HM collected data and was involved in preparation of the manuscript. IA performed the statistical analysis and contributed to preparation of the manuscript. SAS conceived of the study and participated in data interpretation and preparation of the manuscript. All authors read and approved the final manuscript.

## Pre-publication history

The pre-publication history for this paper can be accessed here:

http://www.biomedcentral.com/1472-6939/15/43/prepub
